# Optimization of the Lipase-Catalyzed Selective Amidation of Phenylglycinol

**DOI:** 10.3389/fbioe.2019.00486

**Published:** 2020-01-22

**Authors:** Meina Sun, Kaili Nie, Fang Wang, Li Deng

**Affiliations:** ^1^Beijing Bioprocess Key Laboratory, Beijing University of Chemical Technology, Beijing, China; ^2^Amoy-BUCT Industrial Bio-technovation Institute, Xiamen, China; ^3^State Key Laboratory of Chemical Resource Engineering, Beijing University of Chemical Technology, Beijing, China

**Keywords:** lipase, ceramides analogs, solvent-free, phenylglycinol, amidation

## Abstract

Ceramides and their analogs have a regulatory effect on inflammatory cytokines expression. It was found that a kind of ceramides analog synthesized from phenylglycinol could inhibit the production of cytokine TNF-α. However, two active hydrogen groups are present in the phenylglycinol molecule. It is difficult to control the process without hydroxyl group protection to dominantly produce amide in the traditional chemical synthesis. A selective catalytic the amidation route of phenylglycinol by lipases was investigated in this research. The results indicated that the commercial immobilized lipase Novozym 435 has the best regio-selectivity on the amide group. Based on the experimental results and *in silico* simulation, it was found that the mechanism of specific N-acyl selectivity of lipase was not only from intramolecular migration and proton shuttle mechanism, but also from the special structure of active site of enzyme. The optimal reaction yield of aromatic amide compound in a solvent-free system with lipase loading of 15 wt% (to the weight of total substrate) reached 89.41 ± 2.8% with very few of byproducts detected (0.21 ± 0.1% ester and 0.64 ± 0.2% diacetylated compound). Compare to other reported works, this work have the advantages such as low enzyme loading, solvent free, and high N-acylation selectivity. Meanwhile, this Novozym 435 lipase based synthesis method has an excellent regio-selectivity on most kinds of amino alcohol compounds. Compared to the chemical method, the enzymatic synthesis exhibited high regio-selectivity, and conversion rates. The method could be a promising alternative strategy for the synthesis of aromatic alkanolamides.

**Graphical Abstract:**
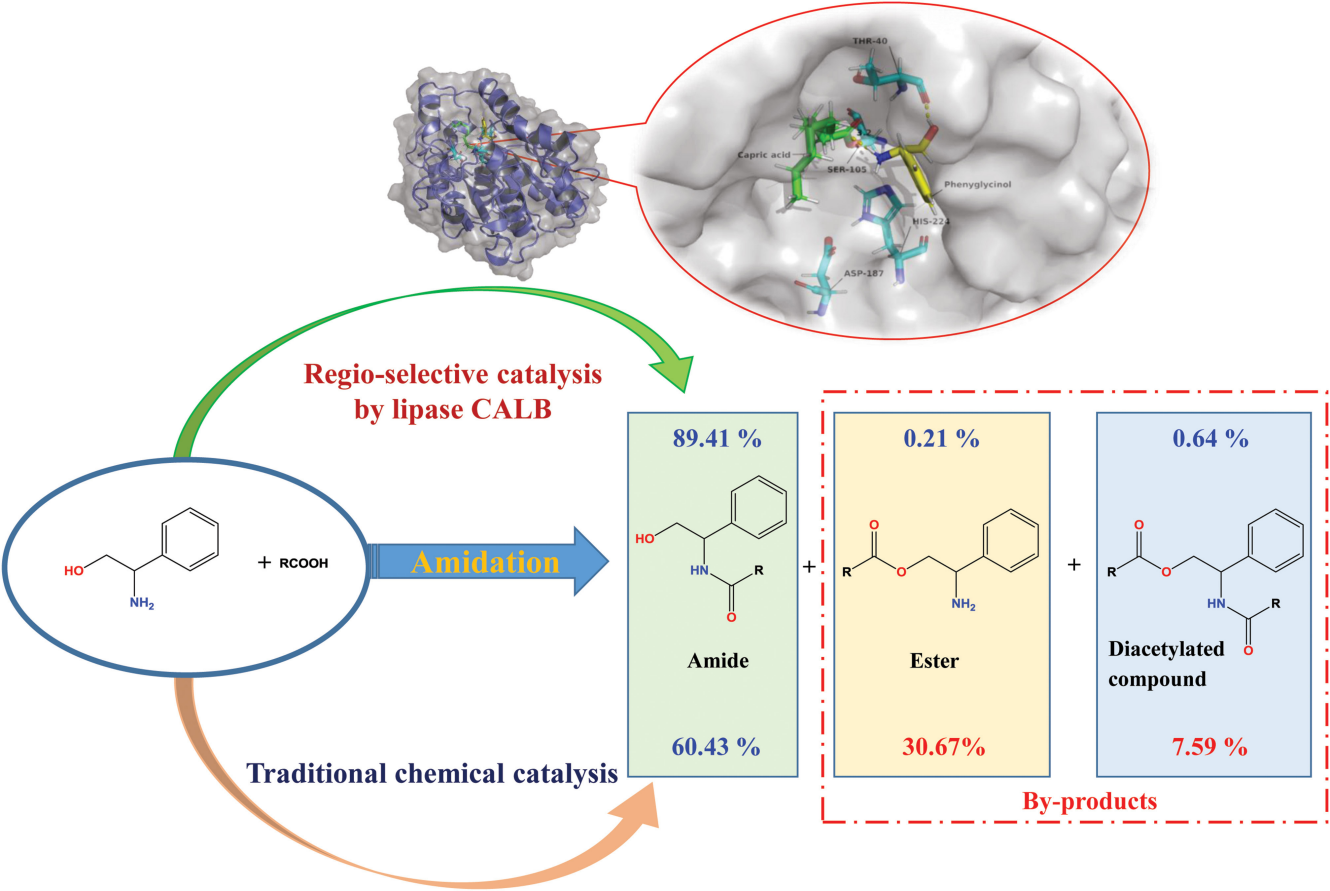
Lipase-catalyzed selective amidation of phenylglycinol.

## Hightlights

- Enzymatic amidation of phenylglycinol and capric acid in solvent-free system.
- Novozyme 435 has effective regio-selectivity for the amidation of phenylglycinol.
- The special structure of enzyme active site influenced regio-selectivity of reaction.
- Enzymatic method is an alternative synthetic strategy for aromatic alkanolamides.

## Introduction

Ceramides are a family of waxy lipid molecules. Normally, they contain a sphingosine and a long chain fatty acid (Long et al., 2006; Sugiyama et al., 2010). Ceramides are found in high concentrations within the cell membrane of eukaryotic cells and within some kinds of plant tissues (Morad and Cabot, 2013). Natural ceramides have a variety of physiological functions, such as their anti-tumor and signal transduction activities, their regulation of apoptosis, their immunity promotion, and their anti-inflammatory properties (Müller et al., 2004). The natural ceramide structure is unstable and difficult to be synthesized.

**Scheme 1:**
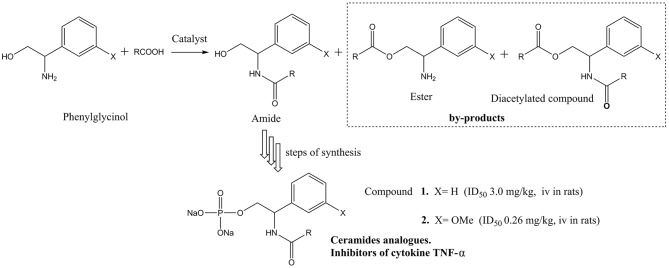
Ceramides analogs synthesize from phenylglycinol.

Over the years, it was found that a series of structure-similar molecules have the physiological activity of ceramides. Parts of those ceramide (Jiang et al., 2016; He and Schuchman, 2018) analogs could act as the inhibiter of cytokines, thus have the potential medicinal properties. Matsui et al. and Toshiaki et al. reported a class of small ceramide analog molecules that could inhibit the production of cytokine TNF-α (Matsui et al., 2002; Toshiaki et al., 2002) (Scheme 1). One of the synthetic routes of compound **1** was used with phenylglycinol as substrate. However, the phenylglycinol molecule contains two active hydrogen groups. It is difficult to control the process without hydroxyl group protection to dominantly produce amide (Andrea et al., 2019). By-products, such as esters and diacetylated compound, would reduce the yield of the amide product.

The traditional chemical catalysis has poor regio-selectivity for the reaction of Scheme 1. It will form a certain amount of by-products. Compared to chemical catalysts, enzymes are more effective toward the substrate specificity and regio-selectivity (Bornscheuer, 2005). Among the enzymes, lipase (EC 3.1.1.3) was often applied for the esterification and amidation reactions (Kirchner et al., 1985; Martinek and Semenov, 2002; Vicente, 2002; Slotema et al., 2003; Tan, 2010). Due to the excellent regio-selectivity, high efficiency and environment amity, many works about lipase, especially commercial lipase Novozym 435, synthesizing high value-added amide products, applied for drug intermediates and cosmetic materials have been reported (Levinson et al., 2005; Martínková et al., 2008; Baum et al., 2011; Schwab et al., 2013; Stavila et al., 2013; Liñares et al., 2014). Among all the researches, amino alcohols have two active hydrogen groups, the amidation reaction on which have been well studied (Syrén et al., 2012; Wang et al., 2015). Based on the results of those researches, it was found that the reactions have outstanding regio-selectivity of N-acylation products. Such as Fernandez-Perez et al. studied the enzymatic synthesis of amide surfactants from ethanolamine, with a good regio-selectivity and yield of 92% N-acyl ethanolamine demonstrated in 1.5 h under the optimal conditions (Fernandez-Perez and Otero, 2001; Irimescu and Kato, 2004). The reason of the regio-selectivity of lipase might be due to two aspects. Firstly, it has been suggested that the lipase-catalyzed N-acylation of amino alcohols proceeded through initial O-acylation followed by spontaneous O- to N-acyl migration (Tufvesson et al., 2006). Secondly, Syrén et al. proposed a proton shuttle reaction mechanism, in which two protons are transferred in the transition state coincidently with the nucleophilic attack, and it would enhance reaction rate for enzyme-catalyzed N-acylation (Syrén et al., 2012).

Even lots of researches have carried out the investigation of lipase catalyzed the N-acylation of amino alcohols, there were few reports about the lipase catalyzed the acylation of phenylglycinol and its similar structure molecules. In this paper, we aimed to utilize lipase to control the selectivity of the amidation of phenylglycinol. The process was optimized and the mechanism of lipase selectivity was explored with the molecular simulation *in silico*. Furthermore, the feasibility of the method for the substrates with the similar structure to phenylglycinol was also investigated.

## Materials and Methods

### Materials

Phenylglycinol and capric acid were obtained from Macleans, Shanghai, China. Bio-catalysts of Novozym435 lipase, Lipozyme RM IM, and Lipozyme TL IM were obtained from Novozymes, Beijing, China. *Candida* sp. 99-125 was purchased from Beijing CTA New Century Biotechnology Co. LTD., Beijing, China, and the lipase was immobilized on diatomaceous earth. The fermentation and immobilization procedure of the lipase were described in our previous research (Tan et al., 2003; Deng et al., 2005). All other chemicals used were of analytical grade, and used as obtained.

### Enzymatic Amidation Reactions

Raw materials of phenylglycinol and capric acid were dissolved for homogeneous mixing at 600 rpm, 78°C, and the substrate molar ratio was set from 1:1 to 1:1.5. After temperature cooling to 60°C, the bio-reaction was carried out for 19 h along with the lipase addition. Moreover, the dosage of the lipase was ranged from 0 to 25 wt% in this reaction. The target production was purified through thin layer chromatography plates and column chromatography that was analyzed by gas chromatography.

### Gas Chromatography

Samples were analyzed by gas chromatography (GC, Shimadzu GC-2010 plus, Japan), which was equipped with FID flame ionization detector. The column was DB-1 capillary column (30 m × 0.25 mm × 0.1 μm, Agilent). Nitrogen with a flow rate of 1.22 mL/min was used as carrier gas for the GC analysis. The column was set at 100°C for 0.2 min, first increased to 220°C at a rate of 8°C/min, then increased to 310°C at a rate of 12°C/min and maintained for 3 min, finally increased to 370°C at a rate of 20°C/min and maintained for 5 min. The injector and the detector temperature were set at 370°C. Each assay was validated at least triplicated for mean values, with margin of error lower than 5%.

### Identification of the Products by FT-IR, ^1^H NMR, and GC-MS

Each component was isolated by crystallization in methanol at −20°C, and the product identification was operated by ^1^H NMR, FT-IR and GC-MS.

### ^1^H NMR

Structure of the purified product was analyzed by ^1^H NMR spectra (NMR, Avance III 400 MHz, Switzerland) with solvent of CDCl_3_. The obtained spectra had verified the product structure with the signals of δ 7.28–7.38 (m, 5H, C_6_H_5_), 6.08 (br s, 1H, NH), 5.05–5.10 (m, 1H, C_6_H_5_ CH), 3.93 (ddd, 2H, CH_2_ OH), 2.61 (br s, 1H, OH), 2.25 (t, 2H, CO CH_2_), 1.60–1.70 (m, 2H), 1.20–1.35 (m, 12H, C_2−7_), 0.88 (m, 3H, C_8_ CH_3_), whose figure had been showed in Figure S1.

### FT-IR

Functionalization of the purified product was analyzed by Fourier transform infrared spectroscopy (FT-IR, Model Nicolet 5ZDX, America) in the range from 400 to 4,000 cm^−1^. The detection results were listed in Figure S2.

### Gas Chromatography Mass Spectrometry

Gas chromatography mass spectra (GC-MS Agilent GC-MS 7890A-5975C) was chosen as the instrument for the product identification. GC-MS analysis was carried out with the column of HP-5 MS capillary column (30 m × 0.25 mm × 0.1 μm, Agilent). Helium was used as carrier gas, with a flow rate of 1.22 mL/min. The column temperature was set at 100°C for 1 min, then increased to 300°C at a rate of 10°C/min, and maintained for 15 min. Mass spectrum was set with ionization voltage of 70 eV, and ion source temperature of 200°C. Assay result was reflected in Figures S3–S23.

### Assay of *in silico* Simulation

The initial opened conformation of YLLIP2, based on the X-ray structure of YLLIP2 (PDB ID: 4JEI), was obtained in our previous study (Cao et al., 2014). Other enzymes were used with their X-ray structure download from PDB protein date bank (http://www.rcsb.org/, *Candida. antarctic* lipase, PDB code: 5GV5; *Thermomyces lanuginosus* lipase, PDB code: 1EIN; *Rhizomucor miehei* lipase, PDB code: 4TGL, respectively). All docking simulations were performed with the YASARA software package (version 16.3.8). AMBER03 force field were used, and docking simulation were carried out at 313 K and atmospheric pressure. Standard docking_run macro (http://www.yasara.org/macros.html) of YASARA was used for simulation. The docking results were viewed and analyzed with PyMOL package (https://pymol.org/).

## Results and Discussion

### Effect of Enzymes on the Amidation Reaction

The modification of only amino group in the substrate molecule is a fundamental challenge of this work. Due to In this work, the conversion rates and regio-selectivity of four kinds of immobilized lipases were investigated.

**Figure 1:**
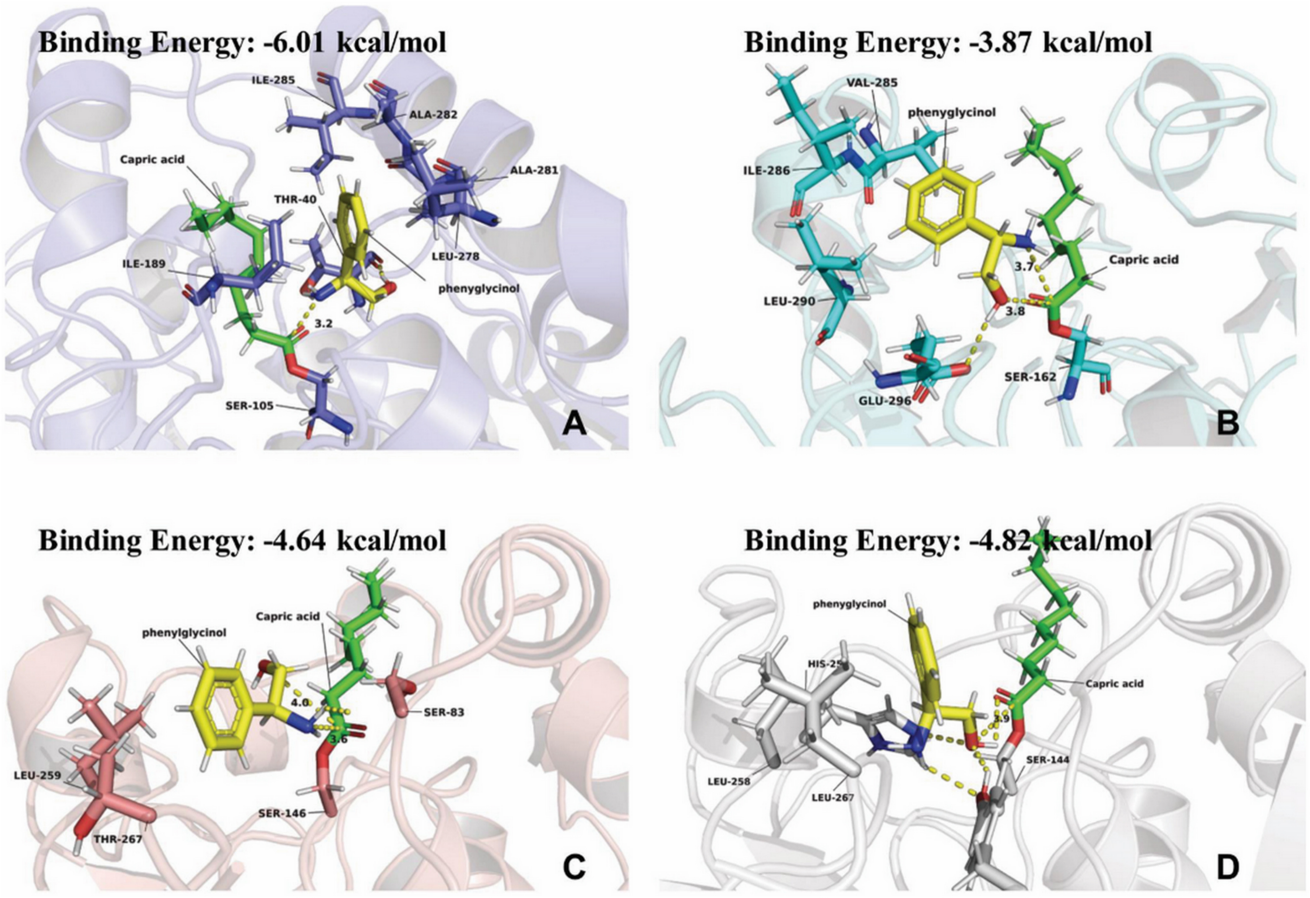
Simulation results of phenylglycinol docking into active site of lipases. **(A)** *Candida. antarctic* lipase B (colored in slate, PDB code: 5GV5). **(B)** *Yarrowia lipolytica* lipase 2 (colored in cyan, PDB code: 4JEI). **(C)** *Thermomyces lanuginosus* lipase (colored in salmon, PDB code: 1EIN). **(D)** *Rhizomucor miehei* lipase (colored in light gray, PDB code: 4TGL). Phenylglycinol was colored in yellow. Capric acid was colored in light green.

The results in Table 1 indicated that compared to other lipases, lipase Novozym 435 (*Candida antarctic* lipase B, CALB) exhibits the best reactivity and regio-selectivity. Normally, when there are more than one functionalities in a substrate which is susceptible to acetylation, the unwanted groups should be protected. However, the results of CALB catalysis (Table 1) indicated that the aim product could be synthesized without group protection. In the early researches about acylation of ethanolamine, Skorey et al. and Tufvesson et al. considered the N-acylation selectivity was through an intramolecular O- to N-acyl migration (Skorey et al., 1987; Tufvesson et al., 2006). However, the migration was based on the balance of thermodynamics (Fodor and Kiss, 1950). It is difficult to explain the reason that different lipases have the various ratios of amide to ester product. Syrén et al. explained the phenomenon as the proton shuttle mechanism (Syrén et al., 2012). Nevertheless, it seemed that the reason was not fit for the results of Lipozyme RM IM (Table 1, line 4). To better understand the mechanism of the different regio-selectivity of various lipases as catalyst in the reaction, a molecular docking process *in silico* was performed. The simulation result of CALB (Figure 1A) indicated that the hydrophobic interaction between the benzene ring and residues of Ile-258, Ala-282, Leu-278, and Ile-189 would maintain the conformation of phenylglycinol. Meanwhile, the hydrogen bonding between the hydroxyl group and Thr-40 makes the amino group close to the active site (Ser-105), and could attack the carbonyl group of the lipase-acylation intermediate (the distance is 3.2 Å). However, the simulation results of *Candida* sp. (*Yarrowia lipolytica* lipase, Figure 1B), and Lipozyme TL IM (*Thermomyces lanuginosus* lipase, Figure 1C) indicated that the hydroxyl and amino group of phenylglycinol have the similar distance to the active site. Whereas, the hydroxyl group of phenylglycinol was much closer to the active site than the amino group in the result of Lipozyme RM IM (*Rhizomucor miehei* lipase, Figure 1D). Thus, those three lipases lacked a regio-selectivity in the reaction. In addition, CALB has the best simulation result of binding energy (−6.01 kcal/mol), which indicated that the active site of CALB lipase is more fit for combination with phenylglycinol than other lipases. Normally, different lipases have specific regio-selectivity due to the special structure of substrate binding domains (Wang et al., 2015). The dominant literatures about lipases catalyzed the acylation of ethanolamine used Novozym 435 (CALB) as catalyst. The protein sequence alignment indicates that the homology of CALB is quite different from other three lipases (Figure S24), and the results of protein structure alignment also verified the structure difference of CALB from others (Figure S25). Based on the results of our experiments and *in silico* docking simulation, it was considered that the mechanism of specific N-acyl selectivity of lipase was not only from intramolecular migration and proton shuttle mechanism, but might be mainly from the special structure of enzyme active site. Therefore, Novozym 435 was chosen as the catalyst for further investigation.

**Table 1:** Effect of different lipases on the amidation reaction.

**Enzymes**	**Phenylglycinol consumption/** **wt%**	**Amide/** **wt%**	**Ester/** **wt%**	**Diacetylated compound/** **wt%**
Novozym 435	41.24 ± 4.32	37.89 ± 3.03	1.42 ± 0.51	1.95 ± 0.64
*Candida* sp. 99–125	27.94 ± 3.20	18.87 ± 4.87	6.16 ± 3.68	3.91 ± 2.10
Lipozyme TL IM	27.11 ± 2.31	18.56 ± 2.68	7.65 ± 2.61	3.43 ± 1.44
Lipozyme RM IM	32.68 ± 4.16	13.35 ± 1.85	16.22 ± 2.42	2.10 ± 1.35

*Reaction conditions: phenylglycinol and capric acid were added at certain molar ratio, the enzyme loading was 10 wt%. The reaction was incubated at 55°C for 10 h with tert-amyl-alcohol as solvent*.

### Effect of Enzyme Amount on the Amidation Reaction

**Figure 2:**
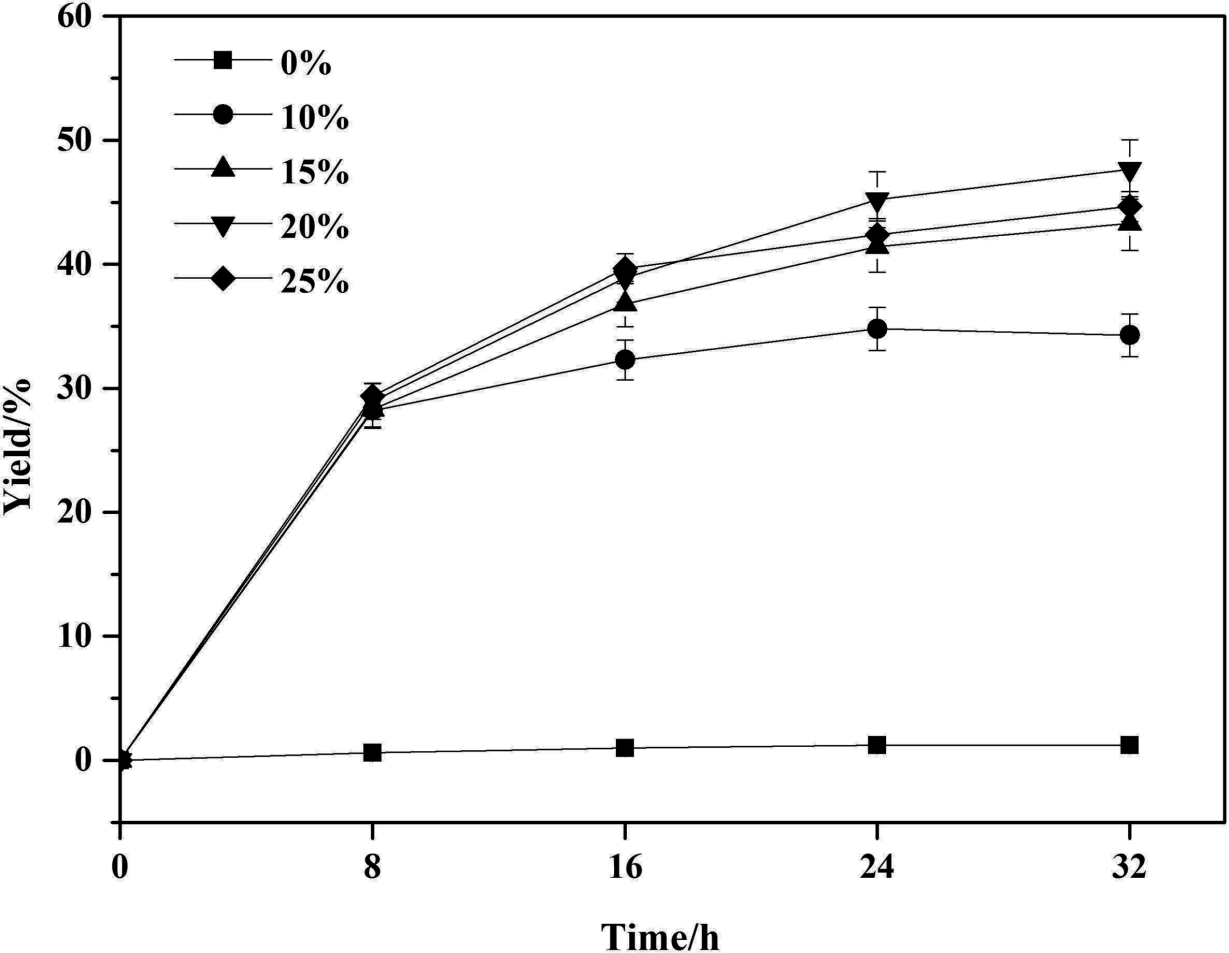
Effect of Novozym 435 amounts on the amidation reaction. Enzymatic reaction conditions: molar ratio of phenylglycinol to capric acid at 1:1, and Novozym 435 loadings varied from 0 to 25 wt%. The reaction was carried out at 40°C for 32 h, with tert-amyl-alcohol as solvent.

The enzyme loading is one of the key factors in the enzymatic reactions. A series of enzyme loadings was carried out and the results were illustrated in Figure 2. Without the addition of enzyme, the amidation reaction of phenylglycinol with capric acid could not be initiated. In the enzymatic groups, the enzyme loadings exhibited no significant differences at the initial 8 h. As the reaction time prolonged, the higher loadings of enzyme facilitated the reaction. When the enzyme loading exceeded 15 wt%, the yield remained stable. This is because of the effective binding of substrates and enzyme active sites would accelerate the reactions. Less or excessive enzyme loadings meant that the availability of active sites of enzymes was inadequate or not properly exposed to the substrates. In addition, excessive enzyme loadings could increase the viscosity of the reaction system, which might prevent efficient mass transfer and result in low catalytic efficiency. Therefore, enzyme loading of 15 wt% is the optimal and economic choice.

### Effect of Solvents and Temperature on the Amidation Reaction

**Figure 3:**
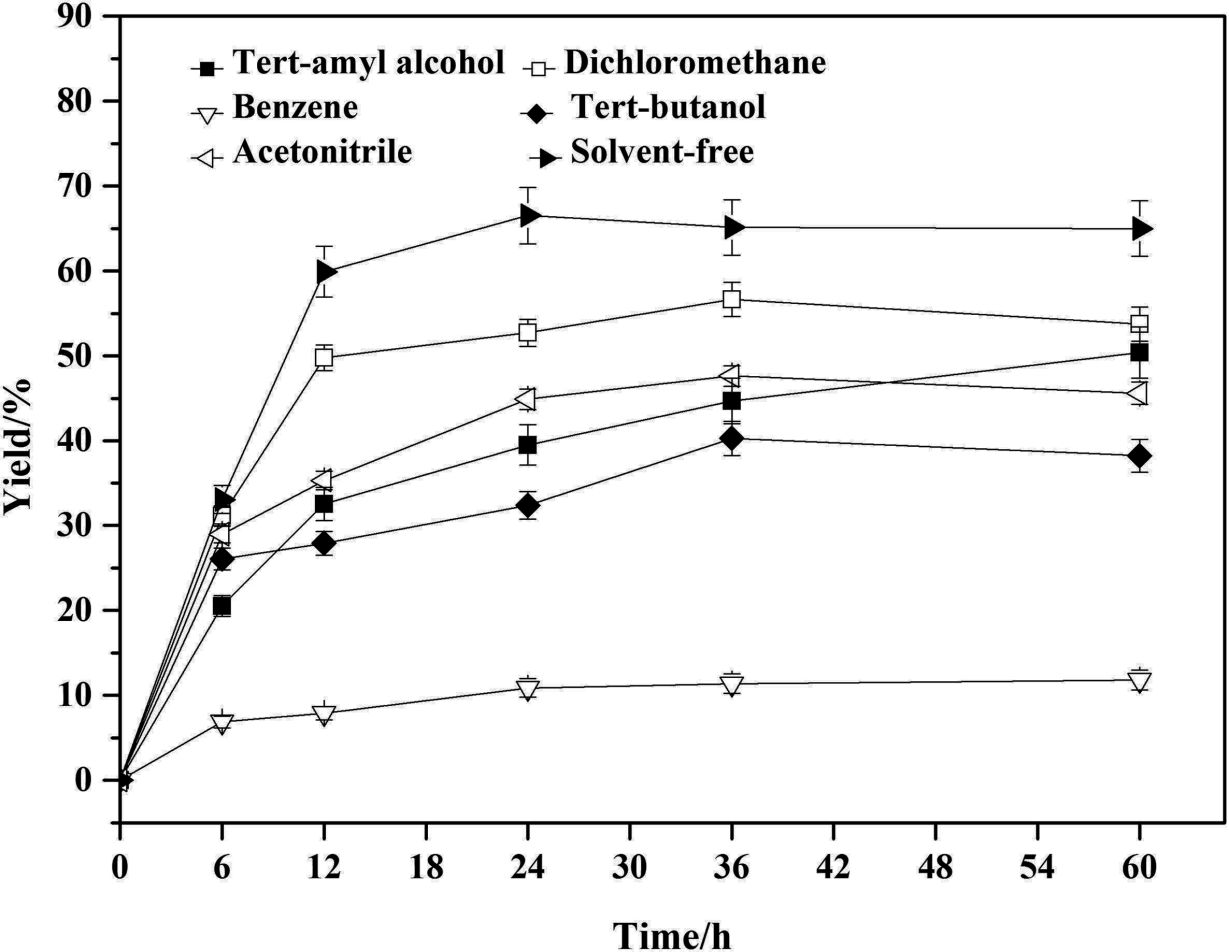
Effect of solvents on the amidation reaction. Enzymatic reaction conditions: molar ratio of phenylglycinol to capric acid at 1:1, and Novozym 435 loading was 15 wt%. The reaction was carried out at 40°C for 60 h.

The role of organic solvents in the enzymatic reaction is to increase the solubility of the substrates and to ensure sufficient contact between the enzyme active sites and the substrates (Fernandez-Perez and Otero, 2003; Levinson et al., 2005; Xiao et al., 2013). However, the organic solvents and temperature also showed great influence on the enzyme activity, stability and selectivity (Zaks and Klibanov, 1984; Ragupathy et al., 2012; Garcia-Alles and Gotor, 2015). The solvent-free system is a greener and safer alternative (Prasad et al., 2005; Ali et al., 2013), but would increase the viscosity of the system. The comparison and selection of solvents was carried out and shown in Figure 3.

A series of organic solvents with different log *P* were screened for the lipase-catalyzed the amidation reactions. Log *P* of benzene, tert-butanol, tert-amyl alcohol, dichloromethane, and acetonitrile were 2.0, 0.6, 1.3, 1.01, and 0.33, respectively. The lower the Log *P-*values, the more hydrophobic the solvents would be. The log *P* of benzene is 2.0, which is beneficial to maintain the activity of the enzyme, but the hydrophobicity is unfavorable to the substrate dissolution. The log *P* of tert-amyl alcohol is 1.3, which is favorable for dissolution of the substrates, but it competes with the essential free water of the enzyme molecule to maintain the catalytic activity, thereby reducing the enzyme activity (Lee and Parkin, 2001; Gorman and Dordick, 2010). Even though the enzymatic synthesis using free fatty acid as acyl donor is a relatively effective process, the amidation reaction conducted in a solvent usually needs 30% (w/w, relative to total reactants) lipase or more (Plastina et al., 2009; Wang et al., 2012, 2013). The high lipase load maybe attributed to the formation of undissolved ion pair from equivalent moles of amine and free fatty acid, and the lipase has a low catalytic activity to ion pair. In the solvent-free system (Prasad et al., 2005), capric acid with the weak polarity could dissolve phenylglycinol completely. This would increase the contact area of the raw material and the enzyme, which would promote the reaction. Therefore we investigated the feasibility of using solvent-free system. The solvent-free system was superior to the applied solvents. Taking the process safety and post-process separation into consideration, the solvent-free system is a better choice. The lipase demonstrates good catalytic efficiency in a solvent-free system. Meanwhile, further investigate about catalyst dosage indicated that 15 wt% was the best of enzyme loading for the solvent-free system (Figure S26).

**Figure 4:**
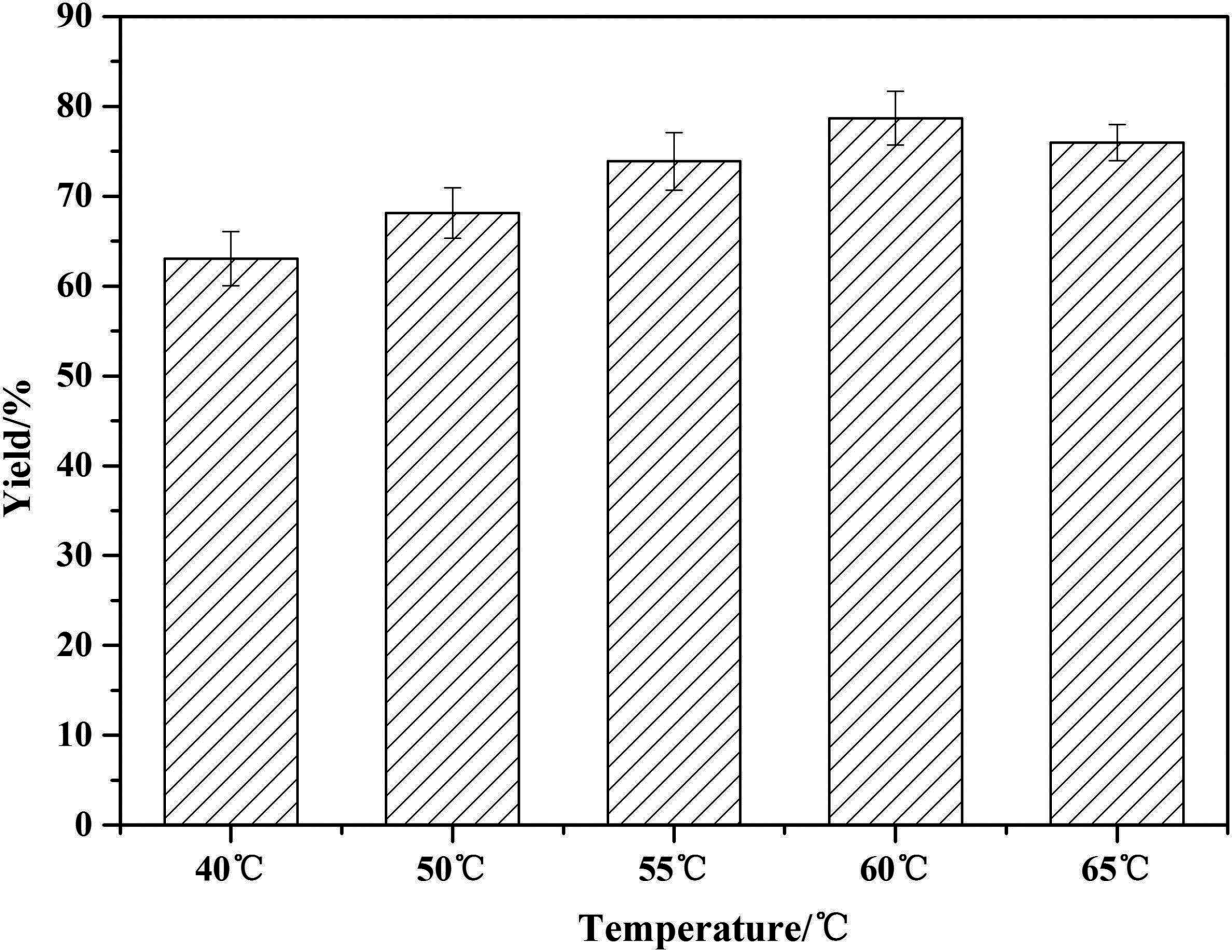
Effect of temperature on the amidation reaction. Enzymatic reaction conditions: a molar ratio of phenylglycinol to capric acid at 1:1, and Novozym 435 loading of 15 wt%. Solvent-free systems for 24 h in a varied but constant temperature mixer.

The optimization of temperature was presented in Figure 4. The lipase activity increases with the increase of reaction temperature in the beginning. The higher temperature can decrease the viscosity of the system, promoting the mass transfer efficiency. The yield increased from 65 to 81% when the temperature rose from 40 to 60°C. As the temperature continued to rise to 65°C, the yield began to decrease. Higher temperature than 60°C led to the denaturation of the enzyme, resulting in a decreased product yield. Therefore, the optimal temperature for solvent-free reaction is 60°C.

### Effect of Substrates Molar Ratio on the Amidation Reaction

**Figure 5:**
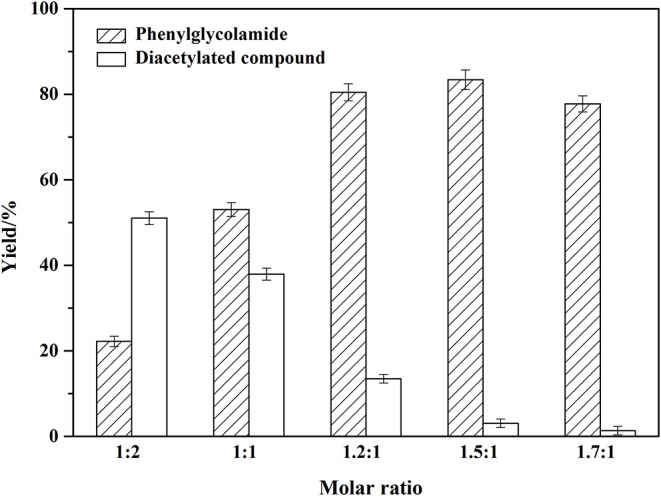
Effect of substrates molar ratio on the amidation reaction. Enzymatic reaction conditions: different molar ratio of phenylglycinol to capric acid; Novozym 435 loading of 15 wt%. Solvent-free systems were carried out at 60°C for 24 h.

Different molar ratios of phenylglycinol to capric acid were investigated (1:2, 1:1, 1.2:1, 1.5:1, 1.7:1) and the results were shown in Figure 5. The ratio of reactants was crucial for selectivity of the enzyme (Maugard et al., 1997). Excess capric acid caused the amino moiety in phenylglycinol to be protonated. Therefore, the amide reaction may be inhibited, resulting in the formation of by-product diacetylated compound. When the molar ratio of phenylglycinol to capric acid increased, the yield of phenylglycolamide increased sharply and by-product diacetylated compound decreased. Excessive phenylglycinol could slow down the formation of ion pairs and inhibit N-acylation (Tufvesson et al., 2006). The N atom of phenylglycinol is less electronegative, while the amino group is more likely to provide electrons. Thus, the amino group has stronger nucleophilic ability than the hydroxyl group, and the activity of the amino hydrogen is stronger than that of hydroxyl group. In this work, it was found that the by-product yield could be controlled from regulation of the substrate molar ratio. The optimal molar ratio of phenylglycinol to capric acid is 1.5:1.

### Effect of Reaction Time on the Amidation Reaction

**Figure 6:**
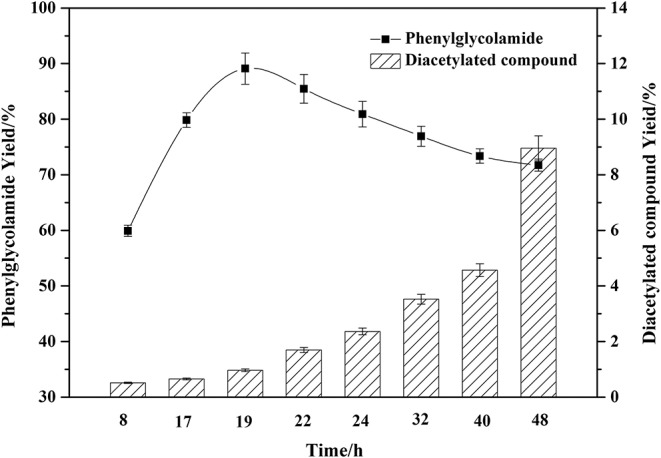
Effect of reaction time on the amidation reaction. Enzymatic reaction conditions: molar ratio of phenylglycinol to capric acid of 1.5:1, and Novozym 435 loading of 15 wt%. Solvent-free reactions at 60°C for different reaction times.

As shown in Figure 6, different reaction times significantly affected the phenylglycolamide yield. The yield of ceramide reached 89.41% at 19 h and then began to decrease. Meanwhile, the by-product diacetylated compound began to accumulate. Reaction times in excess of 19 h might accelerate the consumption of the phenylglycolamide through polymerization or hydrolysis. Thus, 19 h reaction time is adequate for the reaction.

Compared with the previous chemical synthesis of phenylglycolamide, the chemical synthesis of phenylglycolamide required at least 24 h with a yield around 59%. In the present work, the reaction time was reduced to 19 h and the yield was increased to 89.41%. The enzymatic process was superior to the chemical process both in efficiency and environmental amity.

### Enzyme Reusability

**Figure 7:**
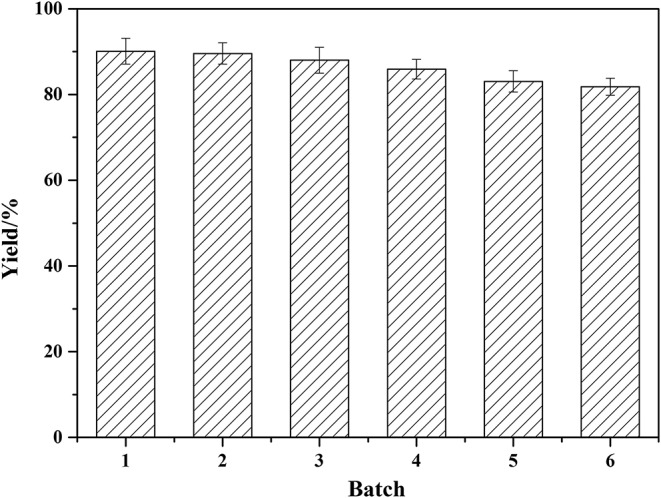
Reusability of Novozym 435. Reaction conditions: molar ratio of phenylglycinol to capric acid of 1.5:1, Novozym 435 loading of 15 wt%. Solvent-free reactions at 60°C for 19 h. Subsequently, the enzyme was washed 3–5 times with hexane, blown dry, and collected for catalysis of the next experiment.

Enzyme reusability was investigated to evaluate the enzyme stability and the results were showed in Figure 7. As demonstrated, Novozym 435 has high catalytic efficiency and good stability under the reaction conditions. After one batch of reaction, the enzyme activity kept stable. Following six batches were repeated, and the yield still maintained about 82%. This proved that the enzymatic process is of good potential for future industrial production.

### The Advantages and Generality of the Method

**Scheme 2:**
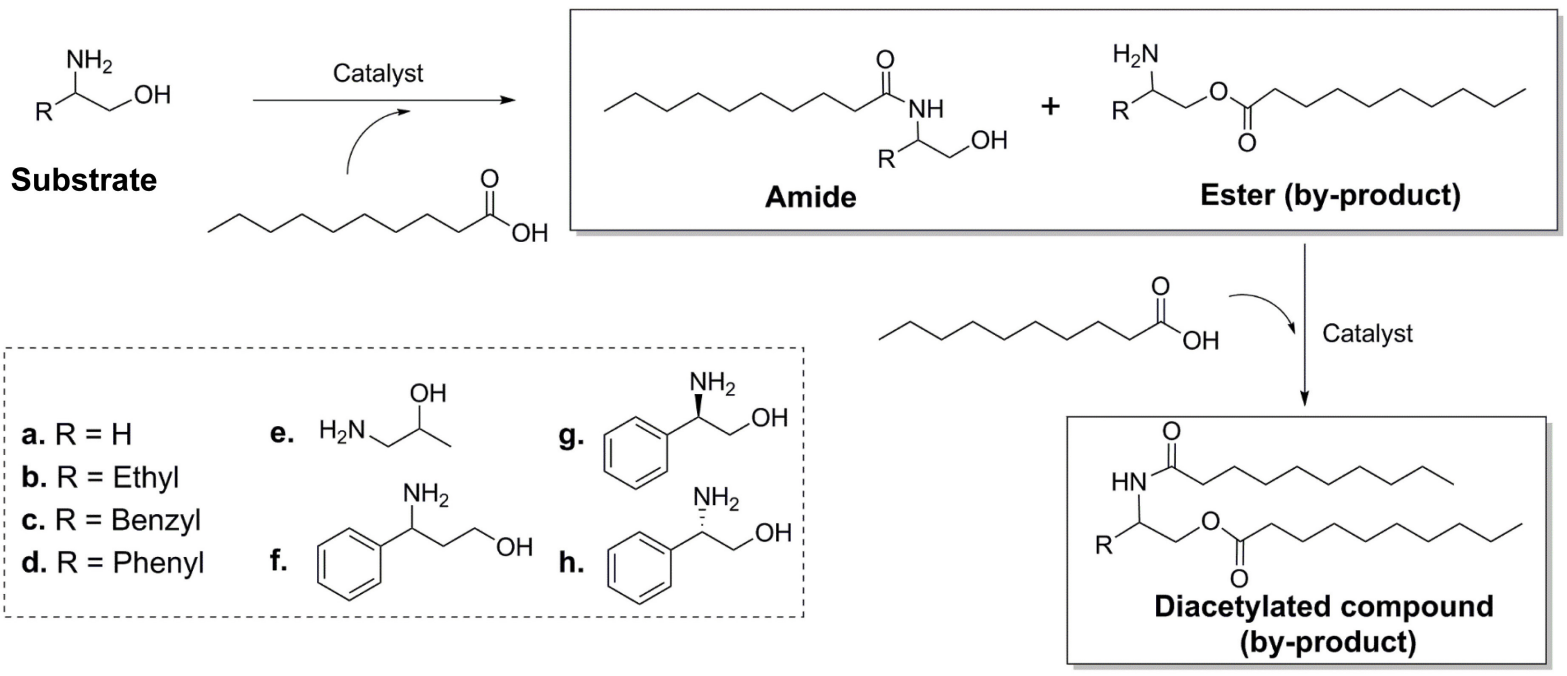
Reaction scheme of amide and by-product formation.

Different kinds of amino alcohols (Scheme 2) were used as substrates to evaluate the generality of the regio-selectivity of lipase. The results (Table 2) indicated that for all kinds of substrates **(a-h)** tested in the experiment, Novozym 435 lipase exhibited specific regio-selectivity to the amino group, and an acceptable conversion. It could also be found that the property of substitute group (-R) would affect the regio-selectivity. Due to the strong electron-pulling effect, among all the substitute group, phenyl group exhibited the best impact. Meanwhile, it was found that CALB enzyme could catalyzed both R-, S-enantiomer of phenylglycinol with similar conversion rates (Table 2, results of g and h), the results is different from other works (Smidt et al., 1996; Irimescu and Kato, 2004), the reason maybe from the chiral resolution need specific solvents (Rios et al., 2008; Vicente and Vicente, 2016), and control the reaction conditions (Joubioux et al., 2013).

**Table 2:** Effect of different methods on the amidation reaction with different kinds of substrate.

**Methods**	**Substrates**	**Amide**/ **wt%**	**Ester/** **wt%**	**Diacetylated compound/** **wt%**
Enzyme catalysis	a	79.68 ± 2.9	8.63 ± 1.3	9.77 ± 1.0
	b	63.68 ± 1.5	3.02 ± 1.0	5.43 ± 0.7
	c	67.73 ± 1.6	1.66 ± 0.4	3.24 ± 0.6
	d^a^	89.41 ± 2.8	0.21 ± 0.1	0.64 ± 0.2
	e	73.60 ± 2.4	4.73 ± 1.2	6.04 ± 2.4
	f	69.58 ± 2.0	0.56 ± 0.1	0.98 ± 0.2
	g^b^	89.32 ± 2.5	0.18 ± 0.09	0.61 ± 0.2
	h^c^	89.35 ± 2.9	0.22 ± 0.1	0.65 ± 0.1
Chemical catalysis	a	61.48 ± 2.3	27.23 ± 1.9	4.27 ± 0.6
	b	56.40 ± 3.0	25.87 ± 1.6	2.19 ± 0.6
	c	53.95 ± 2.5	13.29 ± 1.4	2.05 ± 1.0
	d	60.43 ± 2.6	30.67 ± 1.5	7.59 ± 0.6
	e	59.26 ± 1.2	26.34 ± 1.6	3.68 ± 0.8
	f	55.86 ± 3.0	15.85 ± 1.3	2.86 ± 0.6

a, b, c *The reaction time for the substrate d, g, h were 19 h, for other substrates the reaction time were 24 h. Enzymatic reaction conditions: molar ratio of substrate to capric acid of 1.5:1. Novozym 435 loading of 15 wt%. Solvent-free reactions at 60°C. Chemical reaction conditions: substrate and capric acid were added in a molar ratio of 1.5:1. Sodium methoxide loading was 15 wt%. Methanol was used as solvent. The reaction was carried out in an ice bath for 3 h, then the reaction was continued at room temperature for 24 h*.

The substrate of Table 2 could be divided into two groups. First group is the chain amino alcohols, including **a**, **b**, and **e**. Second group is the aromatic amino alcohols, including **c**, **d**, **f**, **g**, and **h**. For the first group, the results of the reactions were not similar to the results of other literatures. Syrén et al. found that the esters would not be formed in the lipase catalyzed acylation reactions (Syrén et al., 2012). According to our results, a small quantity of ester product was found after 24 h reaction. The reason might attributed to that other researches were carried out in solvents (*tert*-butanol), and in quite short reaction time (10 min), which was not long enough for the accumulation of ester products. For the second group, it was found that CALB illustrated better N-acyl selectivity to the aromatic amino alcohols, and higher reaction conversion rates than chain amino alcohols. This phenomenon might be from two reasons. The products of N-acylation would form the *p-*π conjugate from benzene ring and adjacent amide group, which would increase the stability of product. Meanwhile, from the docking simulation result, it could be found that the benzene ring of substrates would bond at the active site of CALB through hydrophobic interaction, which is benefit for the enzymatic reaction. This also indicated that the structure of lipase is important for the N-acylation selectivity.

The chemical catalysis process with sodium methoxide as catalyst was compared to lipase catalysis. The results of the chemical catalysis process (Table 2) indicated that under the reaction conditions, amide is the main product with all kinds of substrate. This is because of the amide group is more reactive than the hydroxyl group. However, since the chemical catalysis is a homogeneous reaction, the catalyst lacks regio-selectivity, the yield of ester products in chemical catalysis were much higher than that in enzymatic catalysis (Karin et al., 2013; Shirini and Khaligh, 2013; Davis and Phipps, 2017). Moreover, too much side reactions in the chemical catalysis reduced the yield of the amide product to significantly lower values than obtained with enzymatic catalysis.

## Conclusions

In this study, the enzymatic synthesis of aromatic alkanolamides in a solvent free system was investigated. The results indicated that lipase Novozym 435 (lipase CALB) is an efficient catalyst for the reaction. The results of molecular simulation *in silico* illustrated that the specific structure of substrate binding site of lipase CALB determined the regio-selectivity of the lipase. Meanwhile, the results also indicated that the mechanism of specific N-acyl selectivity of lipase was not only from intramolecular migration and proton shuttle mechanism, but also from the special structure of enzyme active site. The optimum conditions for the phenylglycolamide synthesis were determined at a molar ratio of phenylglycinol to capric acid of 1.5:1, an enzyme loading of 15 wt%, a reaction temperature of 60°C, with an agitation speed of 600 rpm for 19 h. Under these optimal reaction conditions, the yield of amide product was 89.41%. The results of evaluation of different amino alcohols as substrate of the enzymatic method proved that the method has an excellent regio-selectivity. Compared to the chemical method, the enzymatic synthesis exhibited high regio-selectivity, and conversions. This is a promising alternative strategy for the synthesis of aromatic alkanolamides.

## Data Availability Statement

All datasets generated for this study are included in the article/Supplementary Material.

## Author Contributions

KN instructed the experiments, and helped to draft, revise the paper, and helped to revise the manuscript. LD designed and revised the experiments. MS performed the experiments and wrote the paper. FW contributed to manuscript revision, read, and approved the submitted version.

### Conflict of Interest

The authors declare that the research was conducted in the absence of any commercial or financial relationships that could be construed as a potential conflict of interest.
