# Supplementary material for: Optimization of the Lipase-Catalyzed Selective Amidation of Phenylglycinol
Source: Front Bioeng Biotechnol. 2020 Jan 22;7:486. doi: 10.3389/fbioe.2019.00486 (PMC6987038; doi:10.3389/fbioe.2019.00486)
Supplement: Supplementary file 1 [file Data_Sheet_1.pdf]

Supplementary material:

Optimization of the lipase-catalyzed selective amidation of phenylglycinol

Meina Sun [1,3], Kaili Nie [1,3 *], Fang Wang [1,2], Li Deng [1,3*]

[1] Beijing Bioprocess Key Laboratory, Beijing University of Chemical Technology, Beijing, China

[2] State Key Laboratory of Chemical Resource Engineering, Beijing University of Chemical Technology, Beijing, China

[3] Amoy-BUCT Industrial Bio-technovation Institute, Xiamen, China

* Correspondences:
Kaili Nie
niekl@mail.buct.edu.cn
Li Deng
dengli@mail.buct.edu.cn

FIGURE CAPTIONS

FIGURE S1. Production assay of the ^{1}H NMR.

^{1}HNMR:(400 MHZ, $CDCl_3$), δ7.28-7.38（m, 5H）, 6.08(br s, 1H), 5.05-5.10(m, 1H), 3.93(ddd, J = 11.0, 6.0, 4.0 HZ 2H), 2.61(brs, 1H), 2.25 (t,J=7.5HZ, 2H), 1.60-1.70 (m, 2H), 1.20-1.35 (m, 12H), 0.88(t, J=7.0HZ, 3H).

FIGURE S2. Assay of the FT-IR spectra.

IR (KBr), v/cm^{-1}:3304, 2926, 1648 (-CH=O), 1540, 1460, 1197, 1039, 700 cm^{-1}.

FIGURE S3. Gas chromatography of compound a.
FIGURE S4. Gas chromatography of compound b.
FIGURE S5. Gas chromatography of compound c.
FIGURE S6. Gas chromatography of compound d.
FIGURE S7. Gas chromatography of compound e.
FIGURE S8. Gas chromatography of compound f

FIGURE S9. Mass spectrometry of amide of compound a.
FIGURE S10. Mass spectrometry of ester of compound a.
FIGURE S11. Mass spectrometry of dimer of compound a.
FIGURE S12. Mass spectrometry of amide of compound b.
FIGURE S13. Mass spectrometry of ester of compound b.
FIGURE S14. Mass spectrometry of dimer of compound b.
FIGURE S15. Mass spectrometry of amide of compound c.
FIGURE S16. Mass spectrometry of ester of compound c.
FIGURE S17. Mass spectrometry of amide of compound d.
FIGURE S18. Mass spectrometry of ester of compound d.
FIGURE S19. Mass spectrometry of dimer of compound d.
FIGURE S20. Mass spectrometry of amide of compound e.
FIGURE S21. Mass spectrometry of ester of compound e.
FIGURE S22. Mass spectrometry of dimer of compound e.
FIGURE S23. Mass spectrometry of amide of compound f.

FIGURE S24. Protein sequence alignment of four kinds of lipases

FIGURE S25. Structure alignment of four kinds of lipase

FIGURE S26. Selection of enzyme amount in organic solvent and solvent-free system

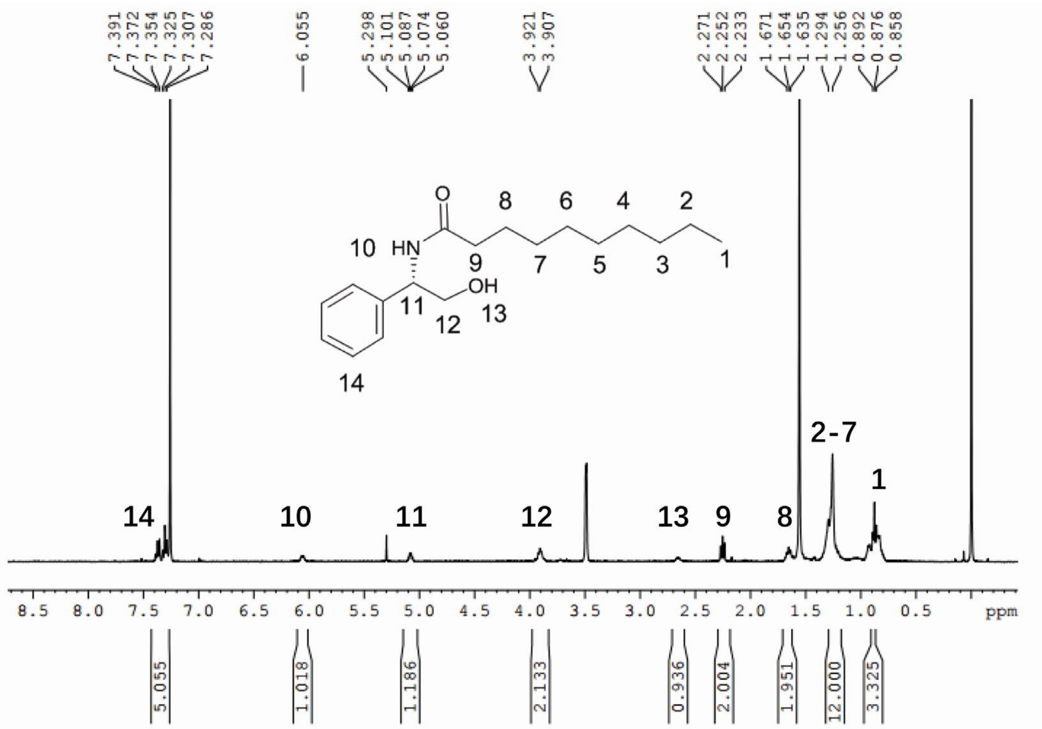

FIGURE S1. Production assay of the ^{1}H NMR.

^{1}HNMR:(400 MHZ, $CDCl_3$), δ7.28-7.38（m, 5H）, 6.08(br s, 1H), 5.05-5.10(m, 1H), 3.93(ddd, J = 11.0, 6.0, 4.0 HZ 2H), 2.61(brs, 1H), 2.25 (t,J=7.5HZ, 2H), 1.60-1.70 (m, 2H), 1.20-1.35 (m, 12H), 0.88(t, J=7.0HZ, 3H).

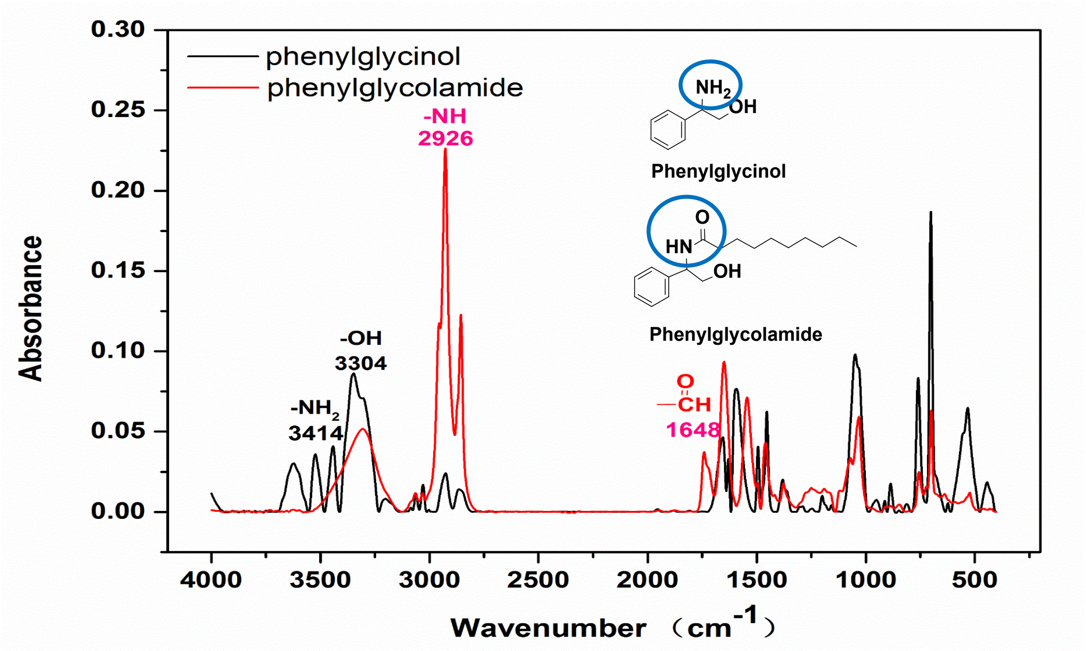

FIGURE S2. Assay of the FT-IR spectra.

IR (KBr), v/cm^{-1}:3304, 2926, 16489（-CH=O）, 1540, 1460, 1197, 1039, 700 cm^{-1}.

a. Ethanolamine

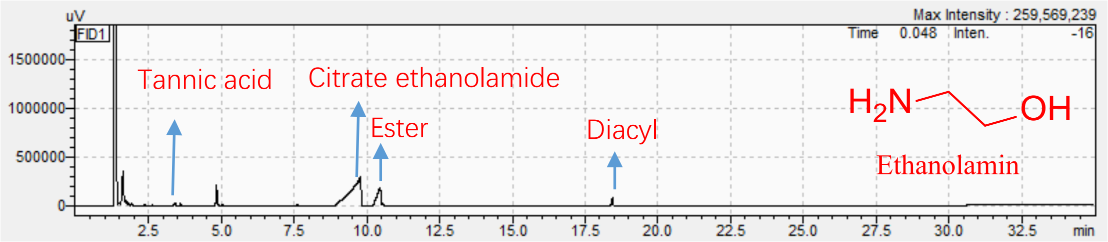

FIGURE S3. Gas chromatography of compound a.

b.2-amino-1-butanol

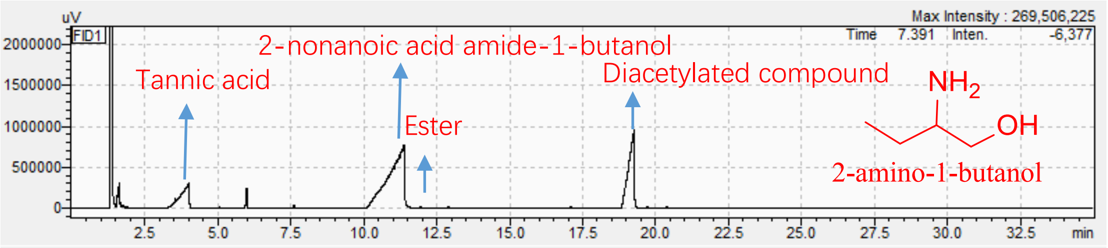

FIGURE S4. Gas chromatography of compound b.

c.Phenylalanine

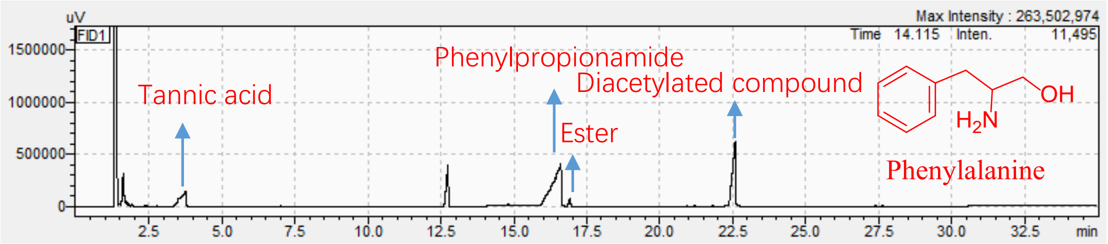

FIGURE S5. Gas chromatography of compound c.

d.Phenylglycinol

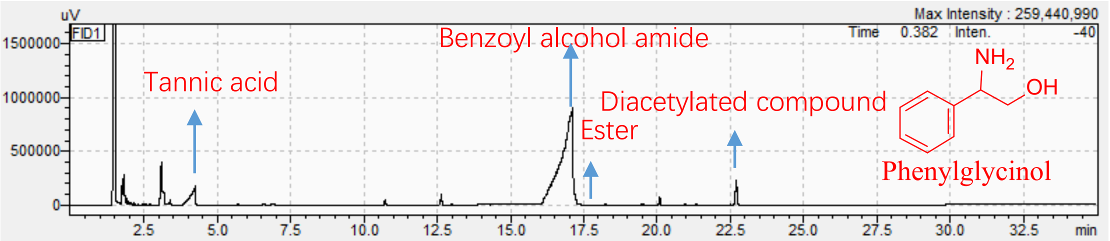

FIGURE S6. Gas chromatography of compound d.

e.Isopropanolamine

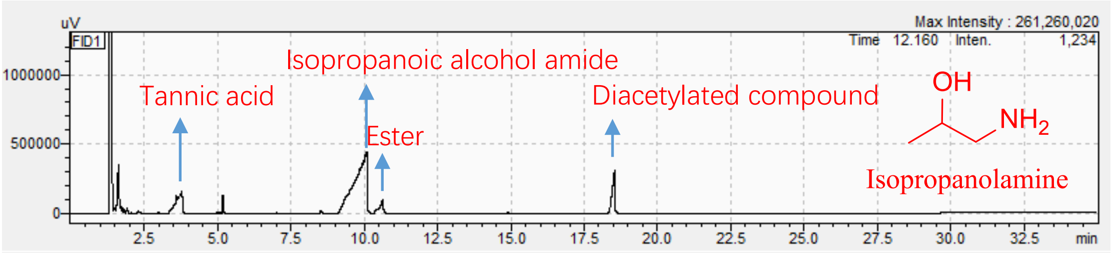

FIGURE S7. Gas chromatography of compound e.

f.3-amino-3-phenyl-1-propanol

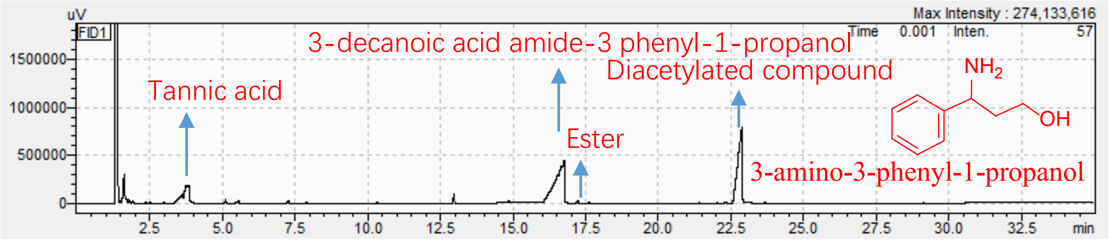

FIGURE S8. Gas chromatography of compound f

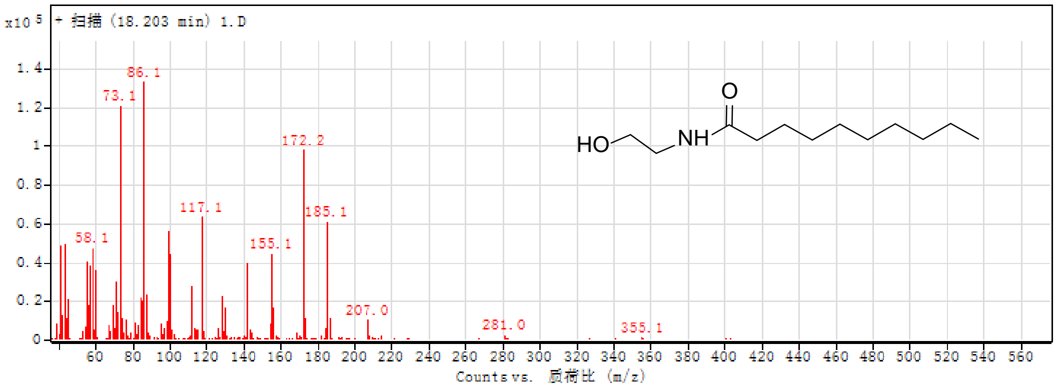

FIGURE S9. Mass spectrometry of amide of compound a.

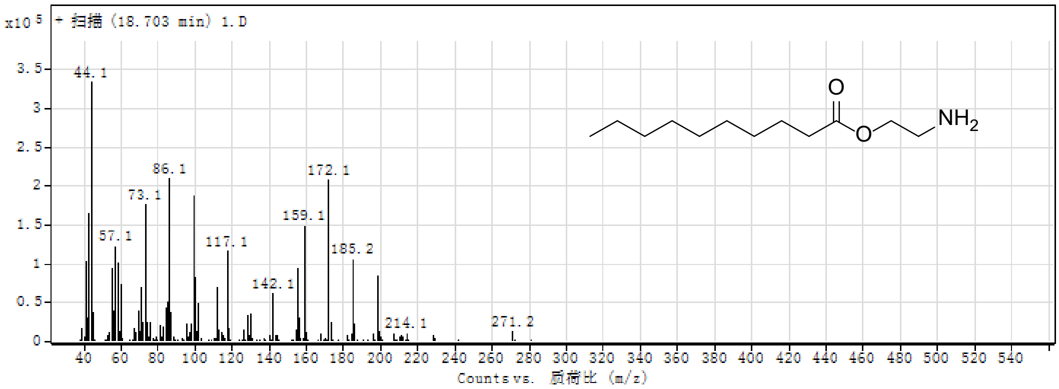

FIGURE S10. Mass spectrometry of ester of compound a.

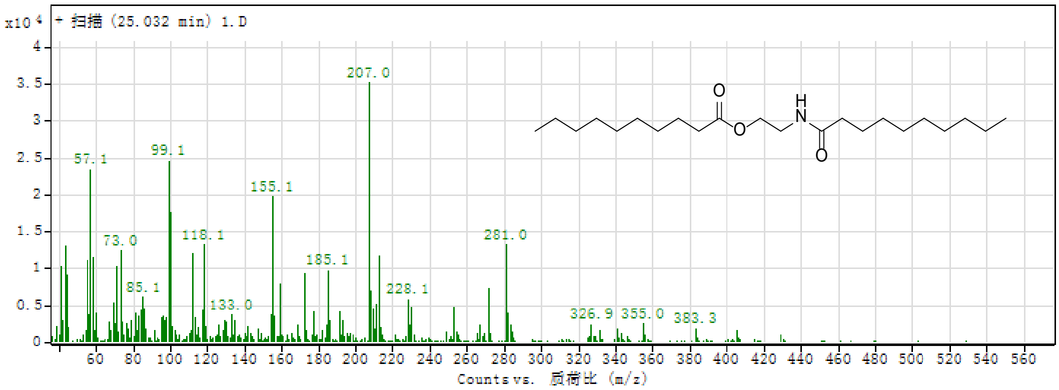

FIGURE S11. Mass spectrometry of dimer of compound a.

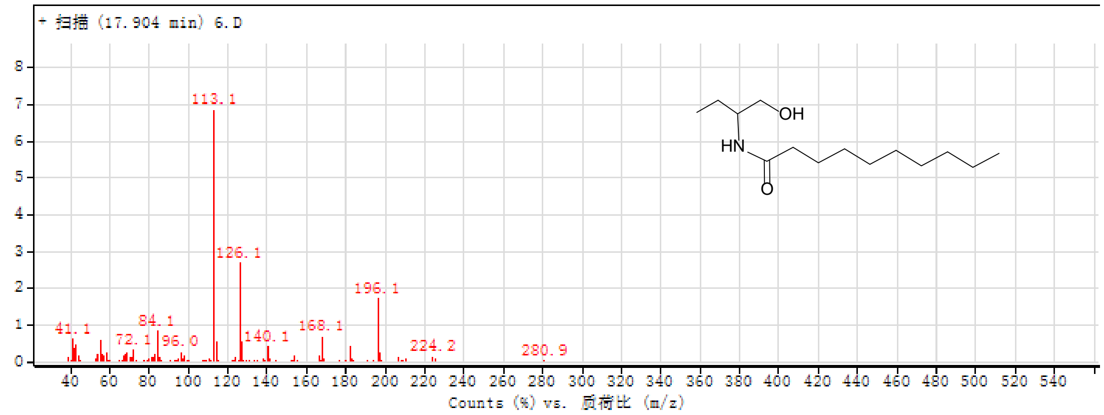

FIGURE S12. Mass spectrometry of amide of compound b.

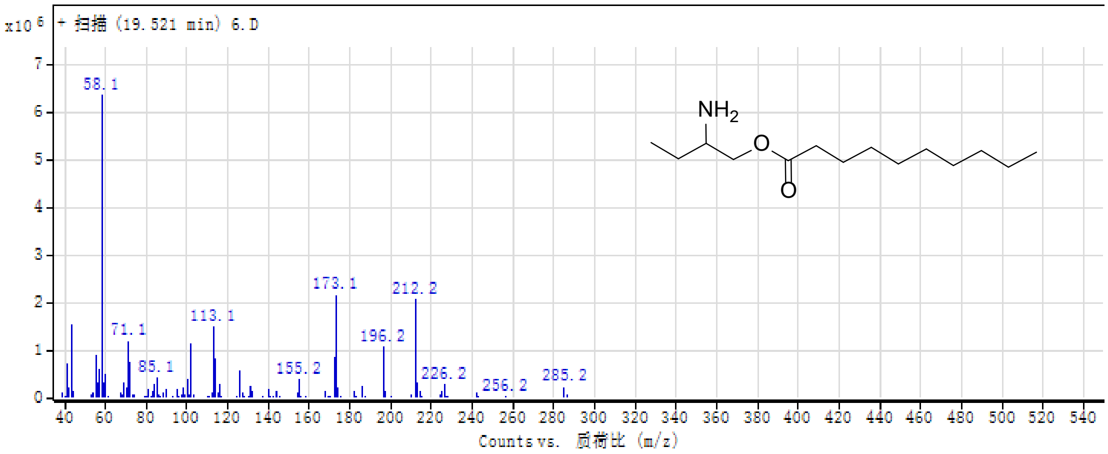

FIGURE S13. Mass spectrometry of ester of compound b.

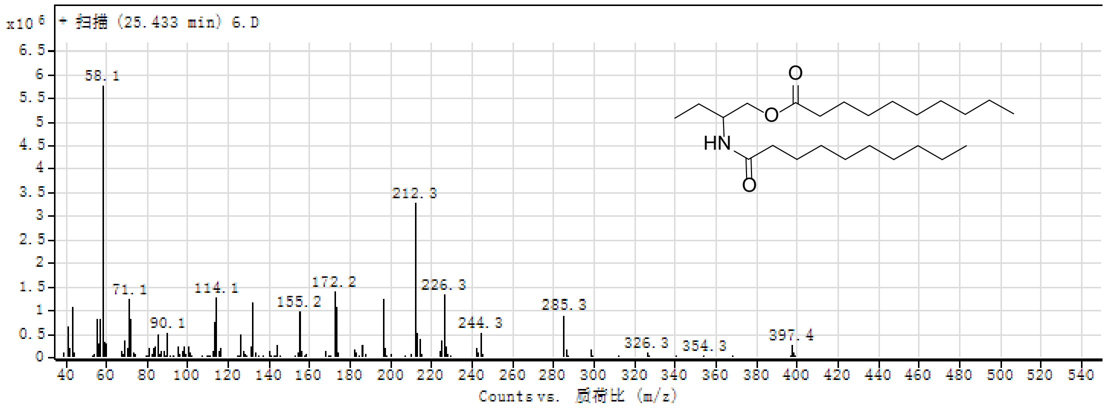

FIGURE S14. Mass spectrometry of dimer of compound b.

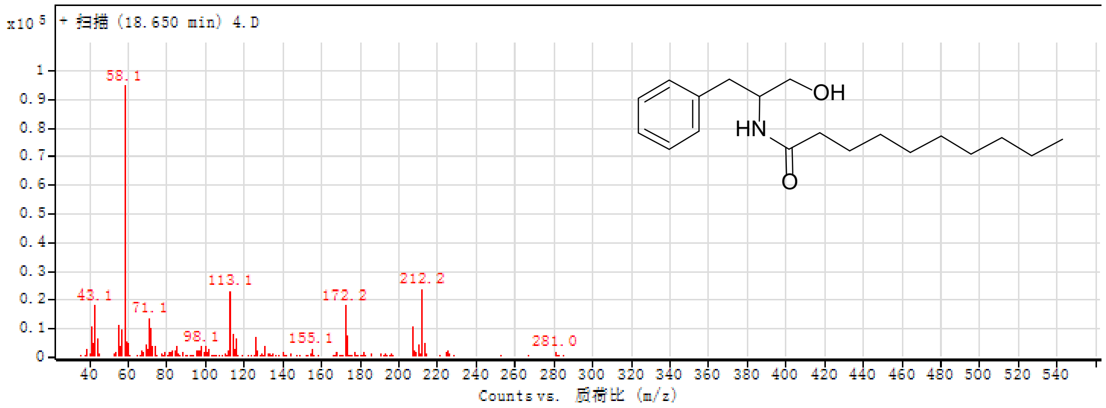

FIGURE S15. Mass spectrometry of amide of compound c.

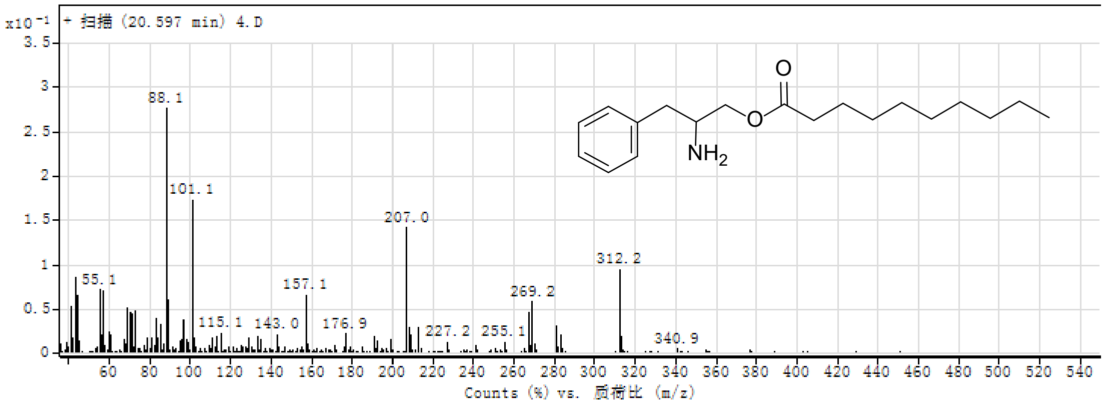

FIGURE S16. Mass spectrometry of ester of compound c.

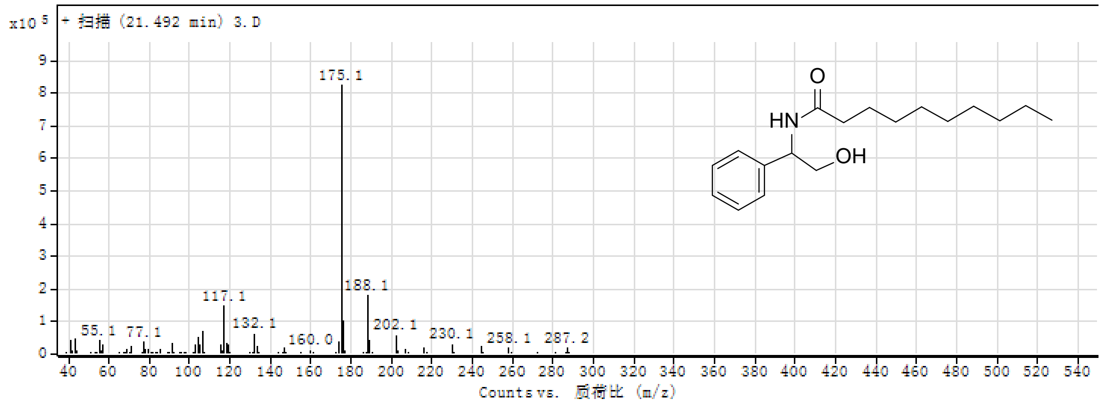

FIGURE S17. Mass spectrometry of amide of compound d.

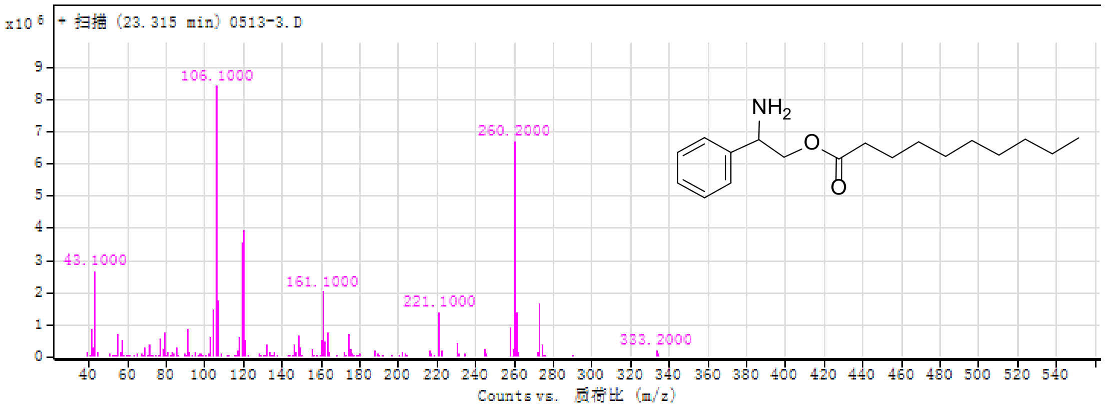

FIGURE S18. Mass spectrometry of ester of compound d.

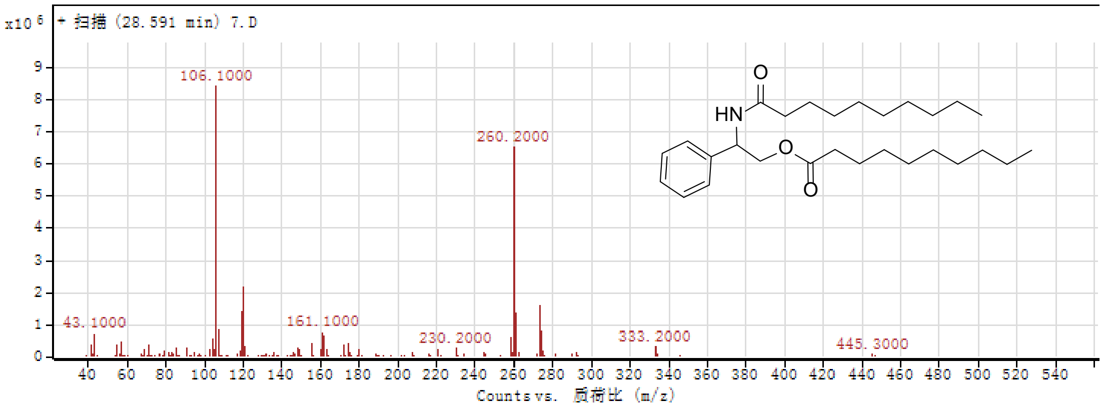

FIGURE S19. Mass spectrometry of dimer of compound d.

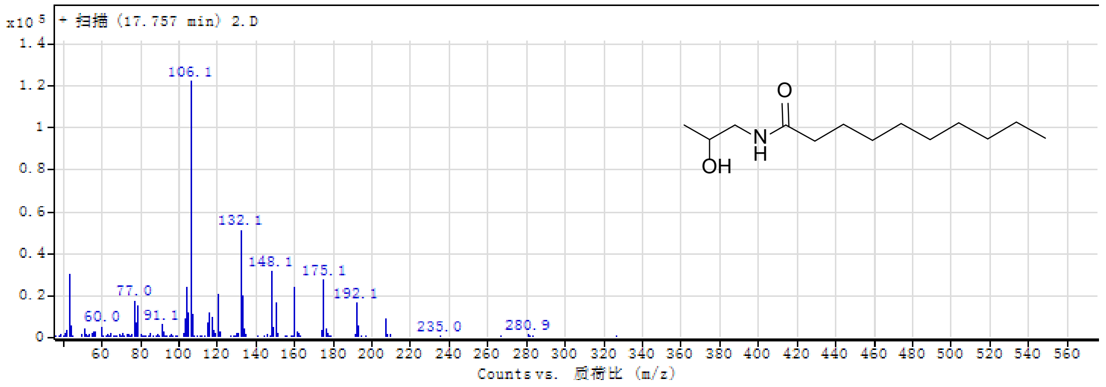

FIGURE S20. Mass spectrometry of amide of compound e.

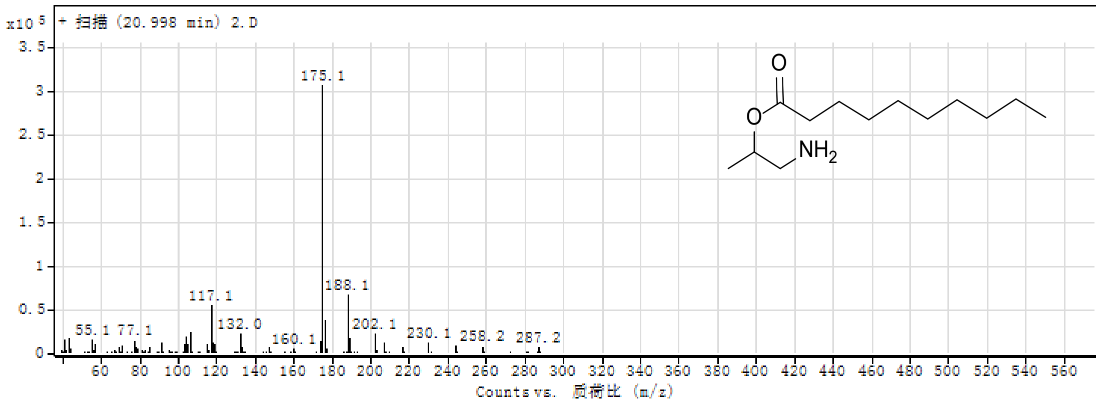

FIGURE S21. Mass spectrometry of ester of compound e.

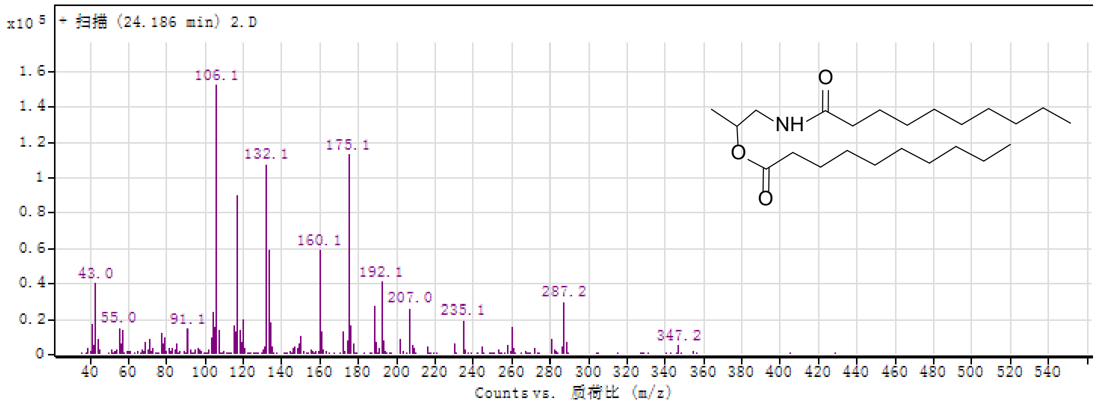

FIGURE S22. Mass spectrometry of dimer of compound e.

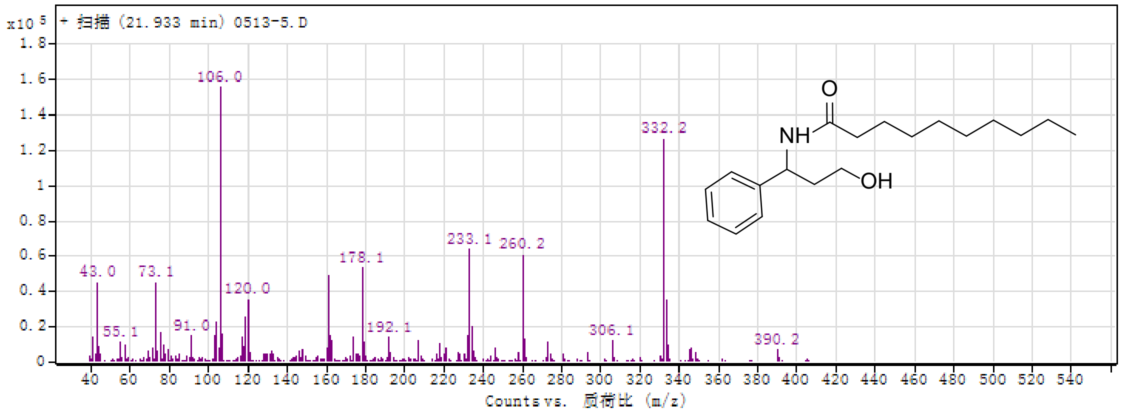

FIGURE S23. Mass spectrometry of amide of compound f.

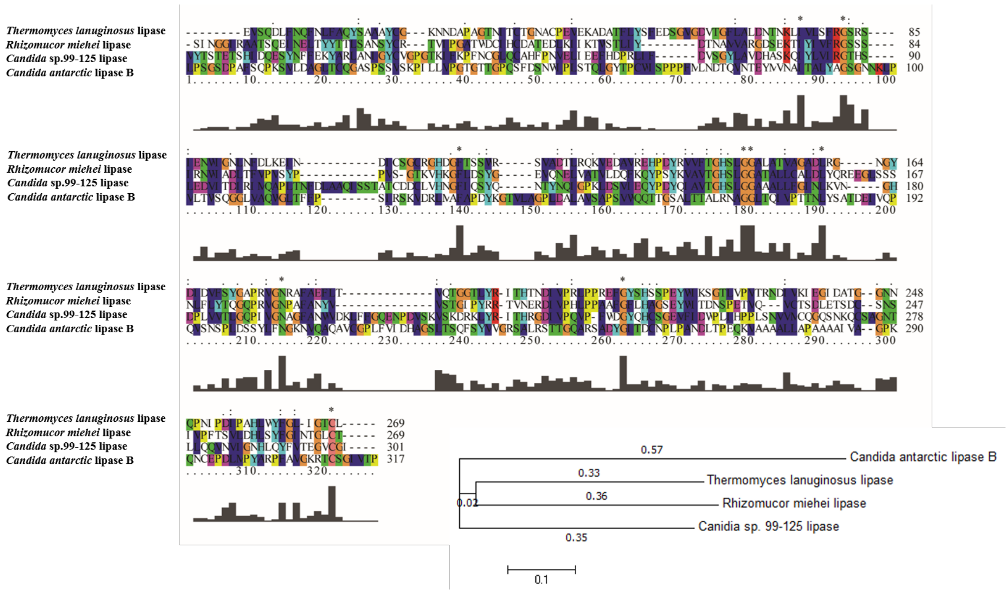

Figure S24 Protein sequence alignment of four kinds of lipases

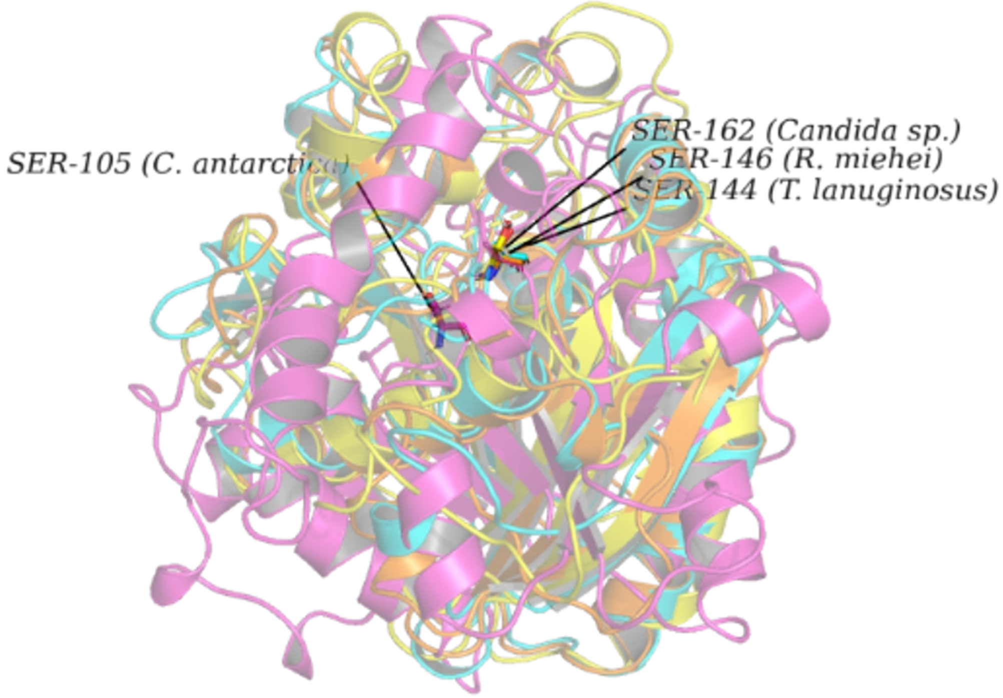

***Thermomyces lanuginosus* lipase, color in cyan; *Rhizomucor miehei* lipase, color in orange; *Candida* sp.99-125 lipase, color in yellow; *Candida antarctic* lipase B; color in purple**

Figure S25 Structure alignment of four kinds of lipase

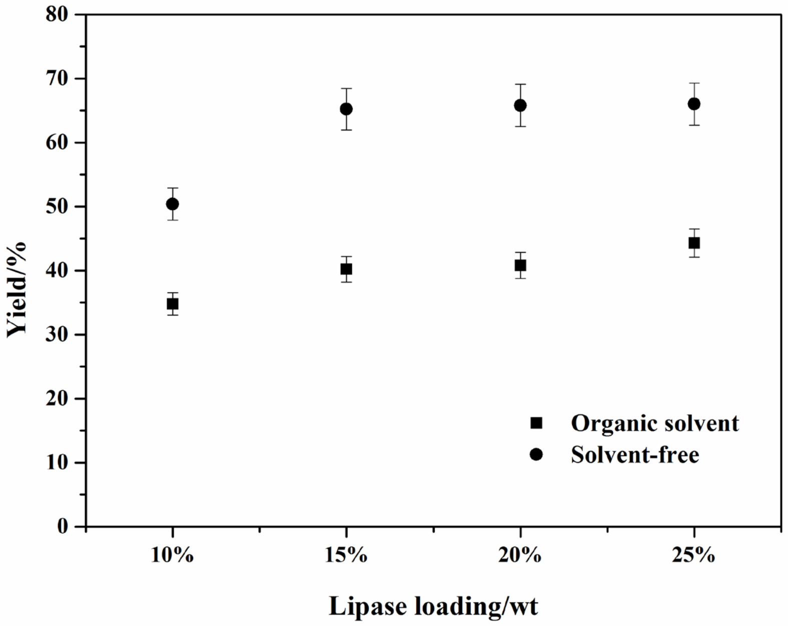

Figure S26 Effect of Novozym 435 amounts on the amidation reaction in organic solvent and solvent-free system.

Figure S26 caption : Effect of Novozym 435 amounts on the amidation reaction in organic solvent and solvent-free system. Enzymatic reaction conditions: molar ratio of phenylglycinol and capric acid at 1:1, and Novozym 435 loadings varied from 10 wt% to 25 wt%. The reaction was carried out at 40 °C for 24 h, separately in organic solvents and solvent-free systems.
